# The effect of social media on the development of students’ affective variables

**DOI:** 10.3389/fpsyg.2022.1010766

**Published:** 2022-09-15

**Authors:** Miao Chen, Xin Xiao

**Affiliations:** ^1^Science and Technology Department, Nanjing University of Posts and Telecommunications, Nanjing, China; ^2^School of Marxism, Hohai University, Nanjing, Jiangsu, China; ^3^Government Enterprise Customer Center, China Mobile Group Jiangsu Co., Ltd., Nanjing, China

**Keywords:** affective variables, education, emotions, social media, post-pandemic, emotional needs

## Abstract

The use of social media is incomparably on the rise among students, influenced by the globalized forms of communication and the post-pandemic rush to use multiple social media platforms for education in different fields of study. Though social media has created tremendous chances for sharing ideas and emotions, the kind of social support it provides might fail to meet students’ emotional needs, or the alleged positive effects might be short-lasting. In recent years, several studies have been conducted to explore the potential effects of social media on students’ affective traits, such as stress, anxiety, depression, and so on. The present paper reviews the findings of the exemplary published works of research to shed light on the positive and negative potential effects of the massive use of social media on students’ emotional well-being. This review can be insightful for teachers who tend to take the potential psychological effects of social media for granted. They may want to know more about the actual effects of the over-reliance on and the excessive (and actually obsessive) use of social media on students’ developing certain images of self and certain emotions which are not necessarily positive. There will be implications for pre- and in-service teacher training and professional development programs and all those involved in student affairs.

## Introduction

Social media has turned into an essential element of individuals’ lives including students in today’s world of communication. Its use is growing significantly more than ever before especially in the post-pandemic era, marked by a great revolution happening to the educational systems. Recent investigations of using social media show that approximately 3 billion individuals worldwide are now communicating via social media (Iwamoto and Chun, 2020). This growing population of social media users is spending more and more time on social network groupings, as facts and figures show that individuals spend 2 h a day, on average, on a variety of social media applications, exchanging pictures and messages, updating status, tweeting, favoring, and commenting on many updated socially shared information (Abbott, 2017).

Researchers have begun to investigate the psychological effects of using social media on students’ lives. Chukwuere and Chukwuere (2017) maintained that social media platforms can be considered the most important source of changing individuals’ mood, because when someone is passively using a social media platform seemingly with no special purpose, s/he can finally feel that his/her mood has changed as a function of the nature of content overviewed. Therefore, positive and negative moods can easily be transferred among the population using social media networks (Chukwuere and Chukwuere, 2017). This may become increasingly important as students are seen to be using social media platforms more than before and social networking is becoming an integral aspect of their lives. As described by Iwamoto and Chun (2020), when students are affected by social media posts, especially due to the increasing reliance on social media use in life, they may be encouraged to begin comparing themselves to others or develop great unrealistic expectations of themselves or others, which can have several affective consequences.

Considering the increasing influence of social media on education, the present paper aims to focus on the affective variables such as depression, stress, and anxiety, and how social media can possibly increase or decrease these emotions in student life. The exemplary works of research on this topic in recent years will be reviewed here, hoping to shed light on the positive and negative effects of these ever-growing influential platforms on the psychology of students.

## Significance of the study

Though social media, as the name suggests, is expected to keep people connected, probably this social connection is only superficial, and not adequately deep and meaningful to help individuals feel emotionally attached to others. The psychological effects of social media on student life need to be studied in more depth to see whether social media really acts as a social support for students and whether students can use social media to cope with negative emotions and develop positive feelings or not. In other words, knowledge of the potential effects of the growing use of social media on students’ emotional well-being can bridge the gap between the alleged promises of social media and what it actually has to offer to students in terms of self-concept, self-respect, social role, and coping strategies (for stress, anxiety, etc.).

### Exemplary general literature on psychological effects of social media

Before getting down to the effects of social media on students’ emotional well-being, some exemplary works of research in recent years on the topic among general populations are reviewed. For one, Aalbers et al. (2018) reported that individuals who spent more time passively working with social media suffered from more intense levels of hopelessness, loneliness, depression, and perceived inferiority. For another, Tang et al. (2013) observed that the procedures of sharing information, commenting, showing likes and dislikes, posting messages, and doing other common activities on social media are correlated with higher stress. Similarly, Ley et al. (2014) described that people who spend 2 h, on average, on social media applications will face many tragic news, posts, and stories which can raise the total intensity of their stress. This stress-provoking effect of social media has been also pinpointed by Weng and Menczer (2015), who contended that social media becomes a main source of stress because people often share all kinds of posts, comments, and stories ranging from politics and economics, to personal and social affairs. According to Iwamoto and Chun (2020), anxiety and depression are the negative emotions that an individual may develop when some source of stress is present. In other words, when social media sources become stress-inducing, there are high chances that anxiety and depression also develop.

Charoensukmongkol (2018) reckoned that the mental health and well-being of the global population can be at a great risk through the uncontrolled massive use of social media. These researchers also showed that social media sources can exert negative affective impacts on teenagers, as they can induce more envy and social comparison. According to Fleck and Johnson-Migalski (2015), though social media, at first, plays the role of a stress-coping strategy, when individuals continue to see stressful conditions (probably experienced and shared by others in media), they begin to develop stress through the passage of time. Chukwuere and Chukwuere (2017) maintained that social media platforms continue to be the major source of changing mood among general populations. For example, someone might be passively using a social media sphere, and s/he may finally find him/herself with a changed mood depending on the nature of the content faced. Then, this good or bad mood is easily shared with others in a flash through the social media. Finally, as Alahmar (2016) described, social media exposes people especially the young generation to new exciting activities and events that may attract them and keep them engaged in different media contexts for hours just passing their time. It usually leads to reduced productivity, reduced academic achievement, and addiction to constant media use (Alahmar, 2016).

The number of studies on the potential psychological effects of social media on people in general is higher than those selectively addressed here. For further insights into this issue, some other suggested works of research include Chang (2012), Sriwilai and Charoensukmongkol (2016), and Zareen et al. (2016). Now, we move to the studies that more specifically explored the effects of social media on students’ affective states.

### Review of the affective influences of social media on students

Vygotsky’s mediational theory (see Fernyhough, 2008) can be regarded as a main theoretical background for the support of social media on learners’ affective states. Based on this theory, social media can play the role of a mediational means between learners and the real environment. Learners’ understanding of this environment can be mediated by the image shaped via social media. This image can be either close to or different from the reality. In the case of the former, learners can develop their self-image and self-esteem. In the case of the latter, learners might develop unrealistic expectations of themselves by comparing themselves to others. As it will be reviewed below among the affective variables increased or decreased in students under the influence of the massive use of social media are anxiety, stress, depression, distress, rumination, and self-esteem. These effects have been explored more among school students in the age range of 13–18 than university students (above 18), but some studies were investigated among college students as well. Exemplary works of research on these affective variables are reviewed here.

In a cross-sectional study, O’Dea and Campbell (2011) explored the impact of online interactions of social networks on the psychological distress of adolescent students. These researchers found a negative correlation between the time spent on social networking and mental distress. Dumitrache et al. (2012) explored the relations between depression and the identity associated with the use of the popular social media, the Facebook. This study showed significant associations between depression and the number of identity-related information pieces shared on this social network. Neira and Barber (2014) explored the relationship between students’ social media use and depressed mood at teenage. No significant correlation was found between these two variables. In the same year, Tsitsika et al. (2014) explored the associations between excessive use of social media and internalizing emotions. These researchers found a positive correlation between more than 2-h a day use of social media and anxiety and depression.

Hanprathet et al. (2015) reported a statistically significant positive correlation between addiction to Facebook and depression among about a thousand high school students in wealthy populations of Thailand and warned against this psychological threat. Sampasa-Kanyinga and Lewis (2015) examined the relationship between social media use and psychological distress. These researchers found that the use of social media for more than 2 h a day was correlated with a higher intensity of psychological distress. Banjanin et al. (2015) tested the relationship between too much use of social networking and depression, yet found no statistically significant correlation between these two variables. Frison and Eggermont (2016) examined the relationships between different forms of Facebook use, perceived social support of social media, and male and female students’ depressed mood. These researchers found a positive association between the passive use of the Facebook and depression and also between the active use of the social media and depression. Furthermore, the perceived social support of the social media was found to mediate this association. Besides, gender was found as the other factor to mediate this relationship.

Vernon et al. (2017) explored change in negative investment in social networking in relation to change in depression and externalizing behavior. These researchers found that increased investment in social media predicted higher depression in adolescent students, which was a function of the effect of higher levels of disrupted sleep. Barry et al. (2017) explored the associations between the use of social media by adolescents and their psychosocial adjustment. Social media activity showed to be positively and moderately associated with depression and anxiety. Another investigation was focused on secondary school students in China conducted by Li et al. (2017). The findings showed a mediating role of insomnia on the significant correlation between depression and addiction to social media. In the same year, Yan et al. (2017) aimed to explore the time spent on social networks and its correlation with anxiety among middle school students. They found a significant positive correlation between more than 2-h use of social networks and the intensity of anxiety.

Also in China, Wang et al. (2018) showed that addiction to social networking sites was correlated positively with depression, and this correlation was mediated by rumination. These researchers also found that this mediating effect was moderated by self-esteem. It means that the effect of addiction on depression was compounded by low self-esteem through rumination. In another work of research, Drouin et al. (2018) showed that though social media is expected to act as a form of social support for the majority of university students, it can adversely affect students’ mental well-being, especially for those who already have high levels of anxiety and depression. In their research, the social media resources were found to be stress-inducing for half of the participants, all university students. The higher education population was also studied by Iwamoto and Chun (2020). These researchers investigated the emotional effects of social media in higher education and found that the socially supportive role of social media was overshadowed in the long run in university students’ lives and, instead, fed into their perceived depression, anxiety, and stress.

Keles et al. (2020) provided a systematic review of the effect of social media on young and teenage students’ depression, psychological distress, and anxiety. They found that depression acted as the most frequent affective variable measured. The most salient risk factors of psychological distress, anxiety, and depression based on the systematic review were activities such as repeated checking for messages, personal investment, the time spent on social media, and problematic or addictive use. Similarly, Mathewson (2020) investigated the effect of using social media on college students’ mental health. The participants stated the experience of anxiety, depression, and suicidality (thoughts of suicide or attempts to suicide). The findings showed that the types and frequency of using social media and the students’ perceived mental health were significantly correlated with each other.

## Discussion

The body of research on the effect of social media on students’ affective and emotional states has led to mixed results. The existing literature shows that there are some positive and some negative affective impacts. Yet, it seems that the latter is pre-dominant. Mathewson (2020) attributed these divergent positive and negative effects to the different theoretical frameworks adopted in different studies and also the different contexts (different countries with whole different educational systems). According to Fredrickson’s broaden-and-build theory of positive emotions (Fredrickson, 2001), the mental repertoires of learners can be built and broadened by how they feel. For instance, some external stimuli might provoke negative emotions such as anxiety and depression in learners. Having experienced these negative emotions, students might repeatedly check their messages on social media or get addicted to them. As a result, their cognitive repertoire and mental capacity might become limited and they might lose their concentration during their learning process. On the other hand, it should be noted that by feeling positive, learners might take full advantage of the affordances of the social media and; thus, be able to follow their learning goals strategically. This point should be highlighted that the link between the use of social media and affective states is bi-directional. Therefore, strategic use of social media or its addictive use by students can direct them toward either positive experiences like enjoyment or negative ones such as anxiety and depression. Also, these mixed positive and negative effects are similar to the findings of several other relevant studies on general populations’ psychological and emotional health. A number of studies (with general research populations not necessarily students) showed that social networks have facilitated the way of staying in touch with family and friends living far away as well as an increased social support (Zhang, 2017). Given the positive and negative emotional effects of social media, social media can either scaffold the emotional repertoire of students, which can develop positive emotions in learners, or induce negative provokers in them, based on which learners might feel negative emotions such as anxiety and depression. However, admittedly, social media has also generated a domain that encourages the act of comparing lives, and striving for approval; therefore, it establishes and internalizes unrealistic perceptions (Virden et al., 2014; Radovic et al., 2017).

It should be mentioned that the susceptibility of affective variables to social media should be interpreted from a dynamic lens. This means that the ecology of the social media can make changes in the emotional experiences of learners. More specifically, students’ affective variables might self-organize into different states under the influence of social media. As for the positive correlation found in many studies between the use of social media and such negative effects as anxiety, depression, and stress, it can be hypothesized that this correlation is induced by the continuous comparison the individual makes and the perception that others are doing better than him/her influenced by the posts that appear on social media. Using social media can play a major role in university students’ psychological well-being than expected. Though most of these studies were correlational, and correlation is not the same as causation, as the studies show that the number of participants experiencing these negative emotions under the influence of social media is significantly high, more extensive research is highly suggested to explore causal effects (Mathewson, 2020).

As the review of exemplary studies showed, some believed that social media increased comparisons that students made between themselves and others. This finding ratifies the relevance of the Interpretation Comparison Model (Stapel and Koomen, 2000; Stapel, 2007) and Festinger’s (1954) Social Comparison Theory. Concerning the negative effects of social media on students’ psychology, it can be argued that individuals may fail to understand that the content presented in social media is usually changed to only represent the attractive aspects of people’s lives, showing an unrealistic image of things. We can add that this argument also supports the relevance of the Social Comparison Theory and the Interpretation Comparison Model (Stapel and Koomen, 2000; Stapel, 2007), because social media sets standards that students think they should compare themselves with. A constant observation of how other students or peers are showing their instances of achievement leads to higher self-evaluation (Stapel and Koomen, 2000). It is conjectured that the ubiquitous role of social media in student life establishes unrealistic expectations and promotes continuous comparison as also pinpointed in the Interpretation Comparison Model (Stapel and Koomen, 2000; Stapel, 2007).

### Implications of the study

The use of social media is ever increasing among students, both at school and university, which is partly because of the promises of technological advances in communication services and partly because of the increased use of social networks for educational purposes in recent years after the pandemic. This consistent use of social media is not expected to leave students’ psychological, affective and emotional states untouched. Thus, it is necessary to know how the growing usage of social networks is associated with students’ affective health on different aspects. Therefore, we found it useful to summarize the research findings in recent years in this respect. If those somehow in charge of student affairs in educational settings are aware of the potential positive or negative effects of social media usage on students, they can better understand the complexities of students’ needs and are better capable of meeting them.

Psychological counseling programs can be initiated at schools or universities to check upon the latest state of students’ mental and emotional health influenced by the pervasive use of social media. The counselors can be made aware of the potential adverse effects of social networking and can adapt the content of their inquiries accordingly. Knowledge of the potential reasons for student anxiety, depression, and stress can help school or university counselors to find individualized coping strategies when they diagnose any symptom of distress in students influenced by an excessive use of social networking.

Admittedly, it is neither possible to discard the use of social media in today’s academic life, nor to keep students’ use of social networks fully controlled. Certainly, the educational space in today’s world cannot do without the social media, which has turned into an integral part of everybody’s life. Yet, probably students need to be instructed on how to take advantage of the media and to be the least affected negatively by its occasional superficial and unrepresentative content. Compensatory programs might be needed at schools or universities to encourage students to avoid making unrealistic and impartial comparisons of themselves and the flamboyant images of others displayed on social media. Students can be taught to develop self-appreciation and self-care while continuing to use the media to their benefit.

The teachers’ role as well as the curriculum developers’ role are becoming more important than ever, as they can significantly help to moderate the adverse effects of the pervasive social media use on students’ mental and emotional health. The kind of groupings formed for instructional purposes, for example, in social media can be done with greater care by teachers to make sure that the members of the groups are homogeneous and the tasks and activities shared in the groups are quite relevant and realistic. The teachers cannot always be in a full control of students’ use of social media, and the other fact is that students do not always and only use social media for educational purposes. They spend more time on social media for communicating with friends or strangers or possibly they just passively receive the content produced out of any educational scope just for entertainment. This uncontrolled and unrealistic content may give them a false image of life events and can threaten their mental and emotional health. Thus, teachers can try to make students aware of the potential hazards of investing too much of their time on following pages or people that publish false and misleading information about their personal or social identities. As students, logically expected, spend more time with their teachers than counselors, they may be better and more receptive to the advice given by the former than the latter.

Teachers may not be in full control of their students’ use of social media, but they have always played an active role in motivating or demotivating students to take particular measures in their academic lives. If teachers are informed of the recent research findings about the potential effects of massively using social media on students, they may find ways to reduce students’ distraction or confusion in class due to the excessive or over-reliant use of these networks. Educators may more often be mesmerized by the promises of technology-, computer- and mobile-assisted learning. They may tend to encourage the use of social media hoping to benefit students’ social and interpersonal skills, self-confidence, stress-managing and the like. Yet, they may be unaware of the potential adverse effects on students’ emotional well-being and, thus, may find the review of the recent relevant research findings insightful. Also, teachers can mediate between learners and social media to manipulate the time learners spend on social media. Research has mainly indicated that students’ emotional experiences are mainly dependent on teachers’ pedagogical approach. They should refrain learners from excessive use of, or overreliance on, social media. Raising learners’ awareness of this fact that individuals should develop their own path of development for learning, and not build their development based on unrealistic comparison of their competences with those of others, can help them consider positive values for their activities on social media and, thus, experience positive emotions.

At higher education, students’ needs are more life-like. For example, their employment-seeking spirits might lead them to create accounts in many social networks, hoping for a better future. However, membership in many of these networks may end in the mere waste of the time that could otherwise be spent on actual on-campus cooperative projects. Universities can provide more on-campus resources both for research and work experience purposes from which the students can benefit more than the cyberspace that can be tricky on many occasions. Two main theories underlying some negative emotions like boredom and anxiety are over-stimulation and under-stimulation. Thus, what learners feel out of their involvement in social media might be directed toward negative emotions due to the stimulating environment of social media. This stimulating environment makes learners rely too much, and spend too much time, on social media or use them obsessively. As a result, they might feel anxious or depressed. Given the ubiquity of social media, these negative emotions can be replaced with positive emotions if learners become aware of the psychological effects of social media. Regarding the affordances of social media for learners, they can take advantage of the potential affordances of these media such as improving their literacy, broadening their communication skills, or enhancing their distance learning opportunities.

## Conclusion

A review of the research findings on the relationship between social media and students’ affective traits revealed both positive and negative findings. Yet, the instances of the latter were more salient and the negative psychological symptoms such as depression, anxiety, and stress have been far from negligible. These findings were discussed in relation to some more relevant theories such as the social comparison theory, which predicted that most of the potential issues with the young generation’s excessive use of social media were induced by the unfair comparisons they made between their own lives and the unrealistic portrayal of others’ on social media. Teachers, education policymakers, curriculum developers, and all those in charge of the student affairs at schools and universities should be made aware of the psychological effects of the pervasive use of social media on students, and the potential threats.

It should be reminded that the alleged socially supportive and communicative promises of the prevalent use of social networking in student life might not be fully realized in practice. Students may lose self-appreciation and gratitude when they compare their current state of life with the snapshots of others’ or peers’. A depressed or stressed-out mood can follow. Students at schools or universities need to learn self-worth to resist the adverse effects of the superficial support they receive from social media. Along this way, they should be assisted by the family and those in charge at schools or universities, most importantly the teachers. As already suggested, counseling programs might help with raising students’ awareness of the potential psychological threats of social media to their health. Considering the ubiquity of social media in everybody’ life including student life worldwide, it seems that more coping and compensatory strategies should be contrived to moderate the adverse psychological effects of the pervasive use of social media on students. Also, the affective influences of social media should not be generalized but they need to be interpreted from an ecological or contextual perspective. This means that learners might have different emotions at different times or different contexts while being involved in social media. More specifically, given the stative approach to learners’ emotions, what learners emotionally experience in their application of social media can be bound to their intra-personal and interpersonal experiences. This means that the same learner at different time points might go through different emotions Also, learners’ emotional states as a result of their engagement in social media cannot be necessarily generalized to all learners in a class.

As the majority of studies on the psychological effects of social media on student life have been conducted on school students than in higher education, it seems it is too soon to make any conclusive remark on this population exclusively. Probably, in future, further studies of the psychological complexities of students at higher education and a better knowledge of their needs can pave the way for making more insightful conclusions about the effects of social media on their affective states.

### Suggestions for further research

The majority of studies on the potential effects of social media usage on students’ psychological well-being are either quantitative or qualitative in type, each with many limitations. Presumably, mixed approaches in near future can better provide a comprehensive assessment of these potential associations. Moreover, most studies on this topic have been cross-sectional in type. There is a significant dearth of longitudinal investigation on the effect of social media on developing positive or negative emotions in students. This seems to be essential as different affective factors such as anxiety, stress, self-esteem, and the like have a developmental nature. Traditional research methods with single-shot designs for data collection fail to capture the nuances of changes in these affective variables. It can be expected that more longitudinal studies in future can show how the continuous use of social media can affect the fluctuations of any of these affective variables during the different academic courses students pass at school or university.

As already raised in some works of research reviewed, the different patterns of impacts of social media on student life depend largely on the educational context. Thus, the same research designs with the same academic grade students and even the same age groups can lead to different findings concerning the effects of social media on student psychology in different countries. In other words, the potential positive and negative effects of popular social media like Facebook, Snapchat, Twitter, etc., on students’ affective conditions can differ across different educational settings in different host countries. Thus, significantly more research is needed in different contexts and cultures to compare the results.

There is also a need for further research on the higher education students and how their affective conditions are positively and negatively affected by the prevalent use of social media. University students’ psychological needs might be different from other academic grades and, thus, the patterns of changes that the overall use of social networking can create in their emotions can be also different. Their main reasons for using social media might be different from school students as well, which need to be investigated more thoroughly. The sorts of interventions needed to moderate the potential negative effects of social networking on them can be different too, all requiring a new line of research in education domain.

Finally, there are hopes that considering the ever-increasing popularity of social networking in education, the potential psychological effects of social media on teachers be explored as well. Though teacher psychology has only recently been considered for research, the literature has provided profound insights into teachers developing stress, motivation, self-esteem, and many other emotions. In today’s world driven by global communications in the cyberspace, teachers like everyone else are affecting and being affected by social networking. The comparison theory can hold true for teachers too. Thus, similar threats (of social media) to self-esteem and self-worth can be there for teachers too besides students, which are worth investigating qualitatively and quantitatively.

Probably a new line of research can be initiated to explore the co-development of teacher and learner psychological traits under the influence of social media use in longitudinal studies. These will certainly entail sophisticated research methods to be capable of unraveling the nuances of variation in these traits and their mutual effects, for example, stress, motivation, and self-esteem. If these are incorporated within mixed-approach works of research, more comprehensive and better insightful findings can be expected to emerge. Correlational studies need to be followed by causal studies in educational settings. As many conditions of the educational settings do not allow for having control groups or randomization, probably, experimental studies do not help with this. Innovative research methods, case studies or else, can be used to further explore the causal relations among the different features of social media use and the development of different affective variables in teachers or learners. Examples of such innovative research methods can be process tracing, qualitative comparative analysis, and longitudinal latent factor modeling (for a more comprehensive view, see Hiver and Al-Hoorie, 2019).

## Author contributions

Both authors listed have made a substantial, direct, and intellectual contribution to the work, and approved it for publication.
